# Supplementary material for: Highly efficient in crystallo energy transduction of light to work
Source: Nat Commun. 2024 Apr 29;15:3633. doi: 10.1038/s41467-024-47881-6 (PMC11059232; doi:10.1038/s41467-024-47881-6)
Supplement: Supplementary file 1 — Supplementary Information [file 41467_2024_47881_MOESM1_ESM.pdf]

Supplementary Information

Highly Efficient *in crystallo* Energy Transduction of Light to Work

Lin et al.

Supplementary Figures

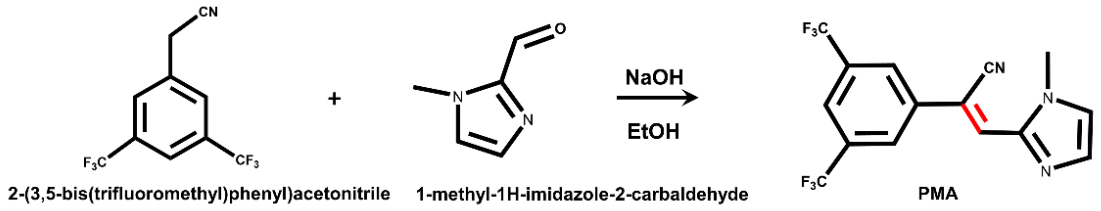

Supplementary Figure 1. Synthetic route for PMA.

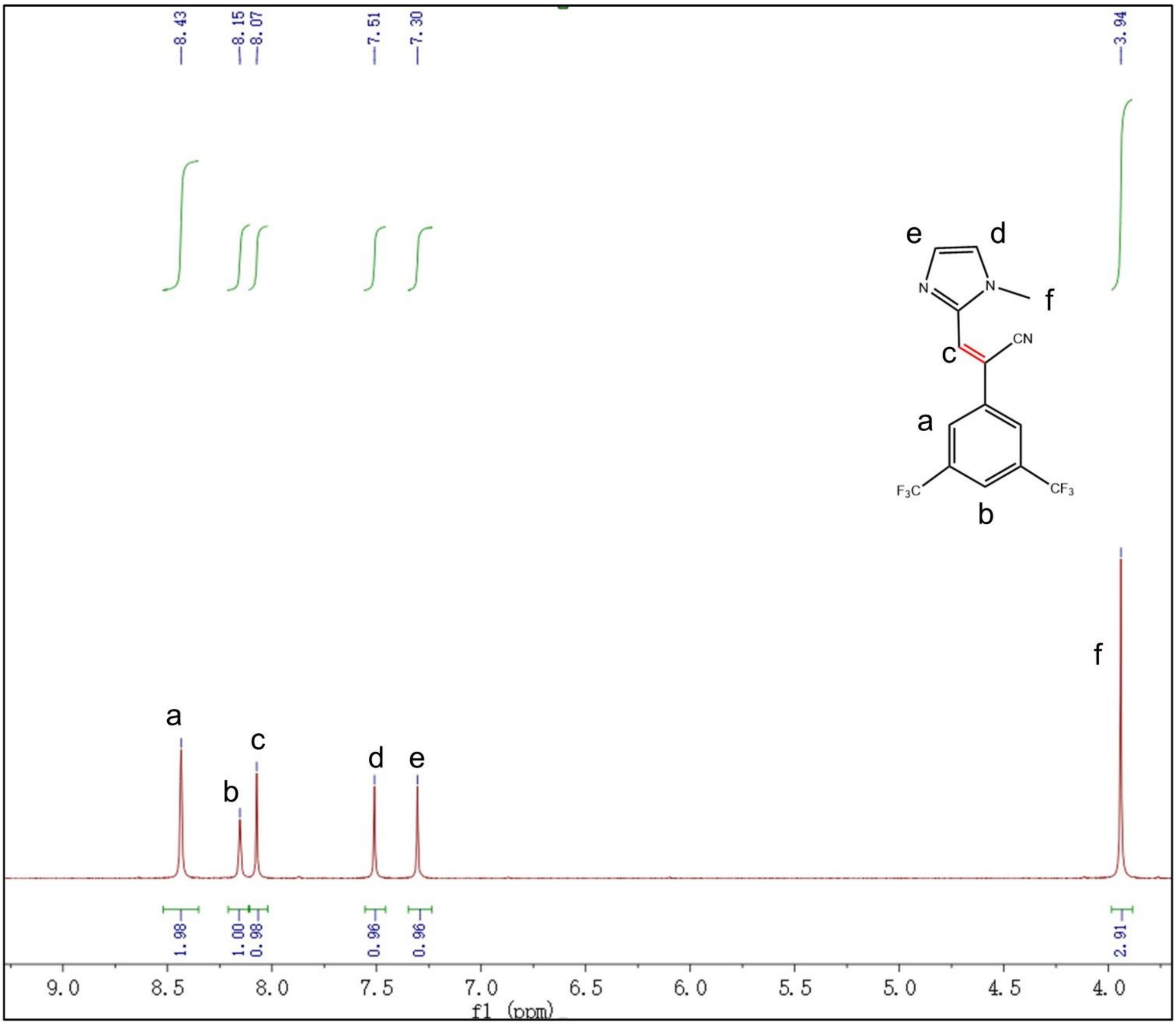

Supplementary Figure 2. ^1H NMR spectrum of PMA in DMSO-d_6 (400 MHz). ^1H NMR (400 MHz, DMSO-d6) δ/ppm 8.43 (s, 2H), 8.15 (s, 1H), 8.07 (s, 1H), 7.51 (s, 1H), 7.30 (s, 1H), 3.94 (s, 3H).

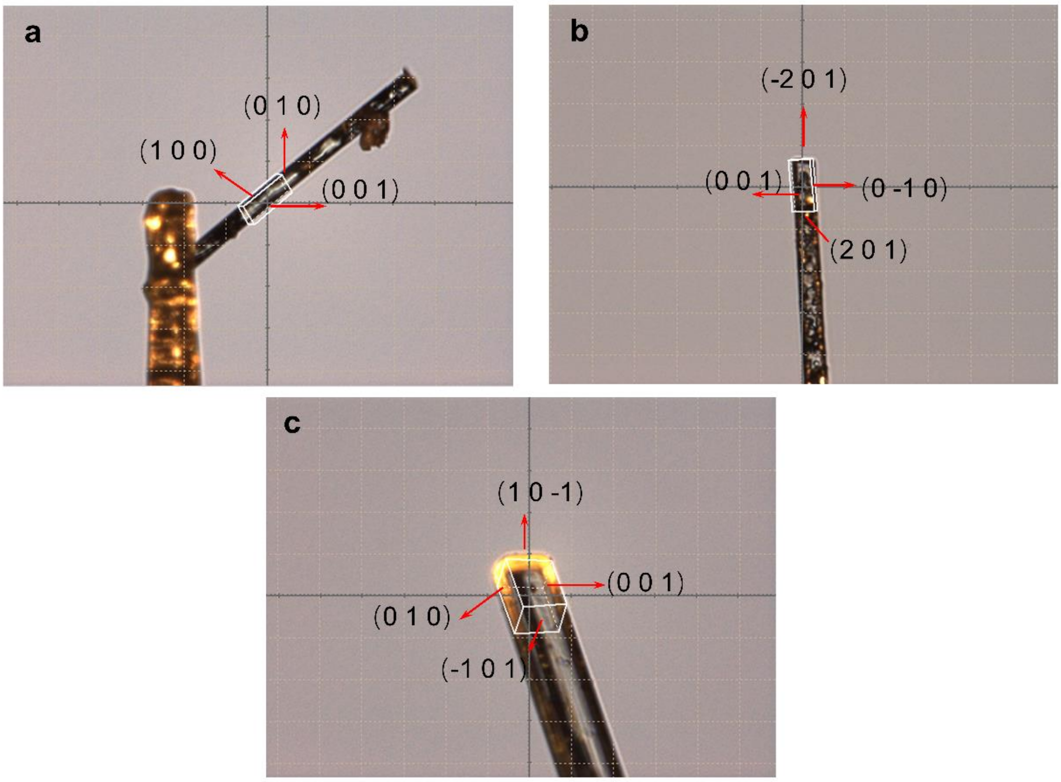

Supplementary Figure 3. Face indexing of three polymorphs. (a) PMA-I. (b) PMA-II. (c) PMA-III.

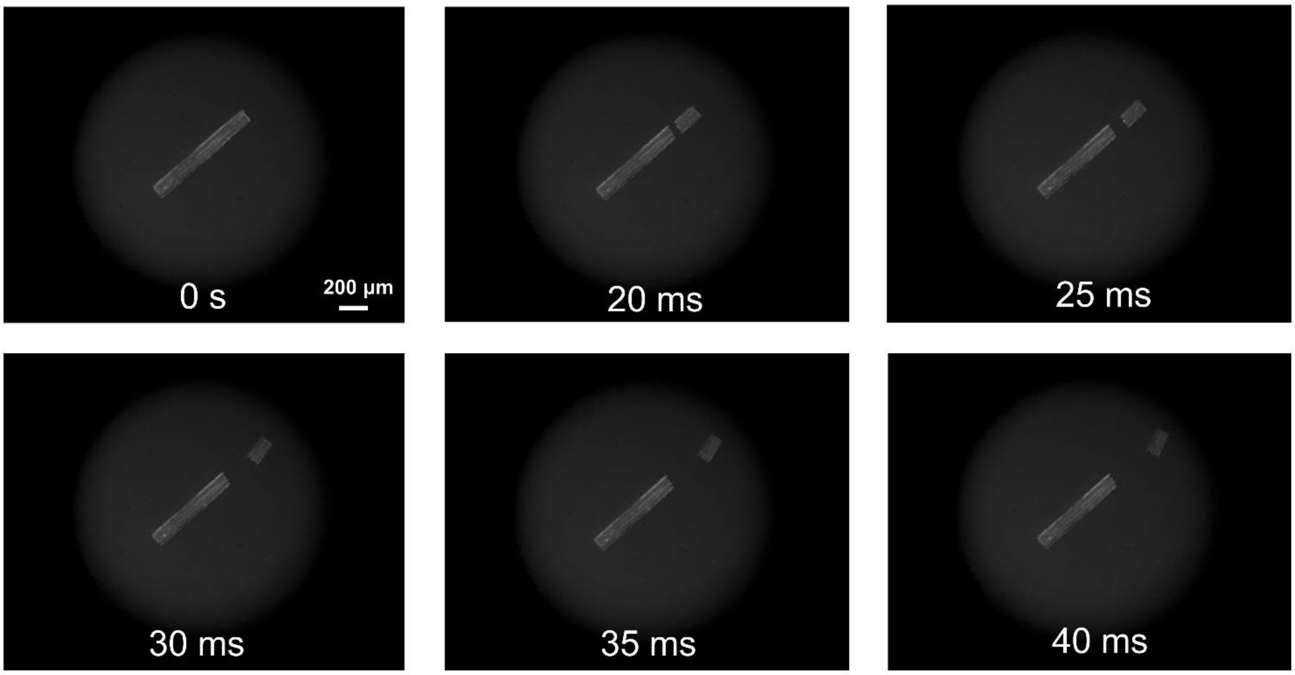

Supplementary Figure 4. Snapshots extracted from optical high-speed video recordings of the photosalient behaviors of bulk single crystals of PMA-I.

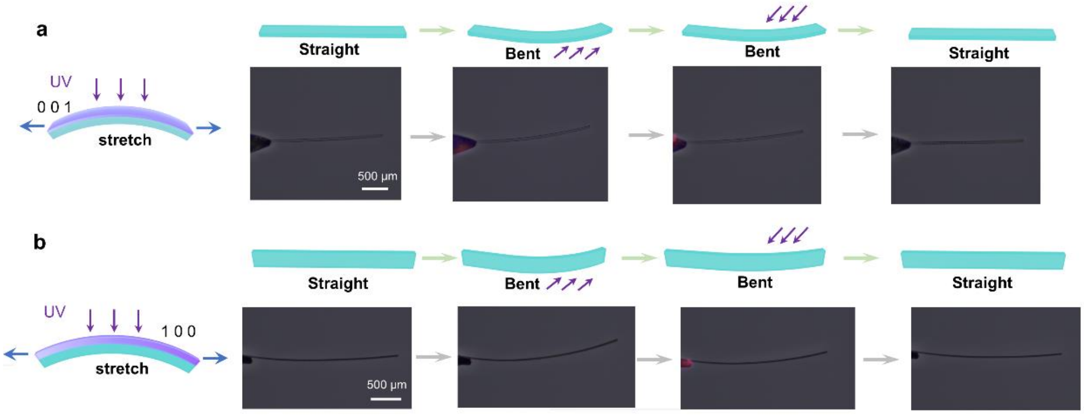

Supplementary Figure 5. Photoinduced bending of PMA-I. (a) Bending along the (001) plane. (b) Bending along the (100) plane.

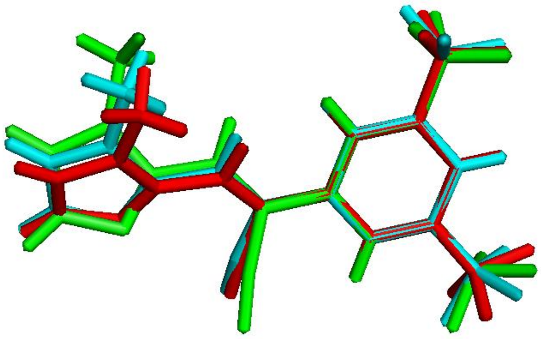

Supplementary Figure 6. Overlaid representation of the molecules from the three polymorphs. Green: PMA-I, blue: PMA-II, red: PMA-III.

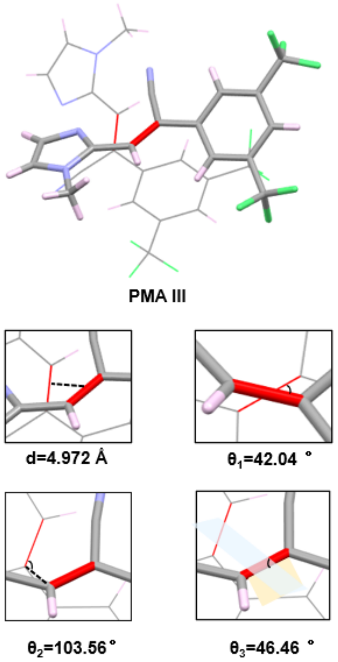

Supplementary Figure 7. Molecular packing of PMA-III and parameters relevant to the lack of reactivity of the crystals.

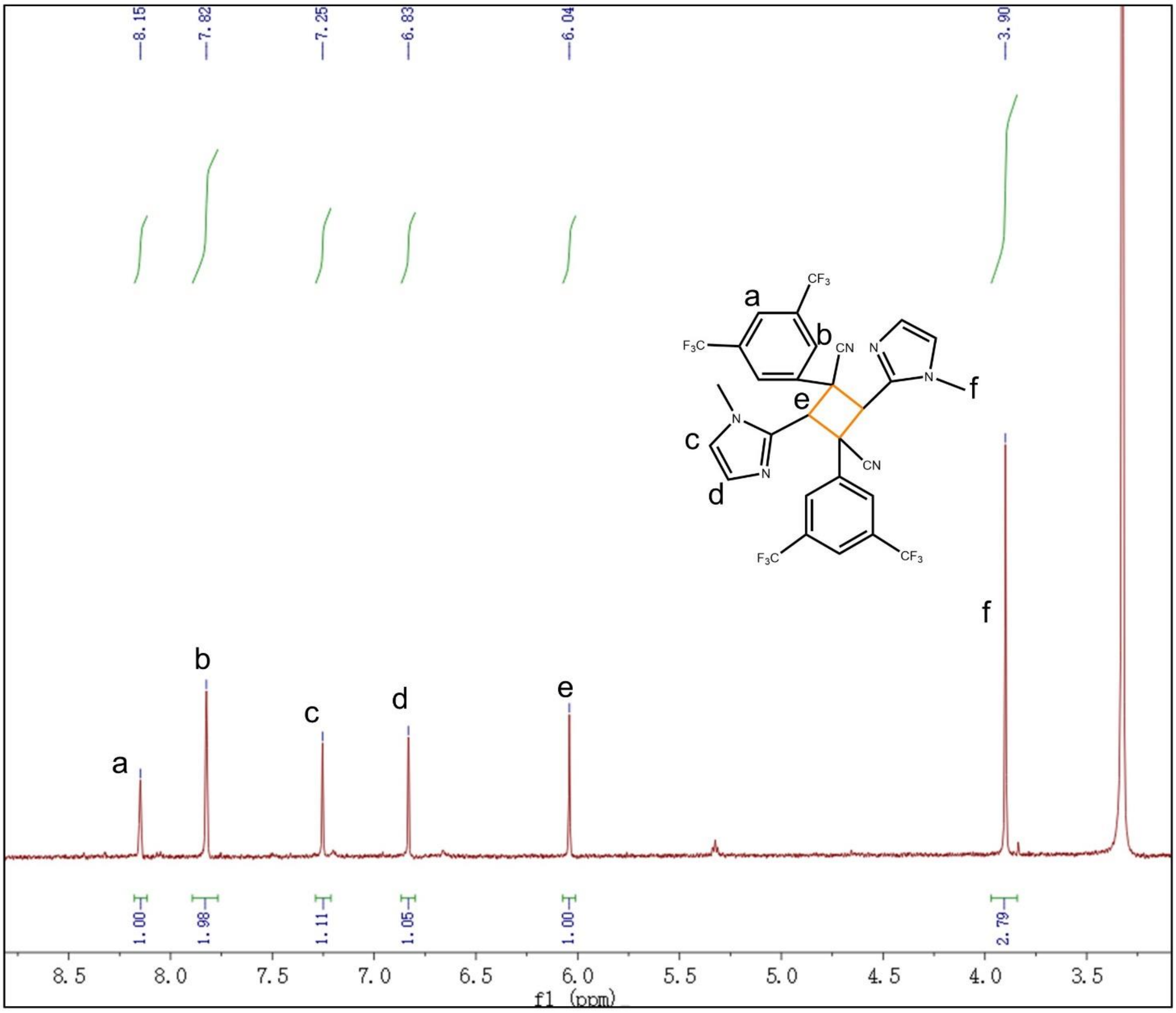

Supplementary Figure 8. ^{1}HNMR spectra of TMC. ^{1}H NMR (400 MHz, DMSO) δ/ppm 8.15 (s, 2H), 7.82 (s, 4H), 7.25 (s, 2H), 6.83 (s, 2H), 6.04 (s, 2H), 3.90 (s, 6H).

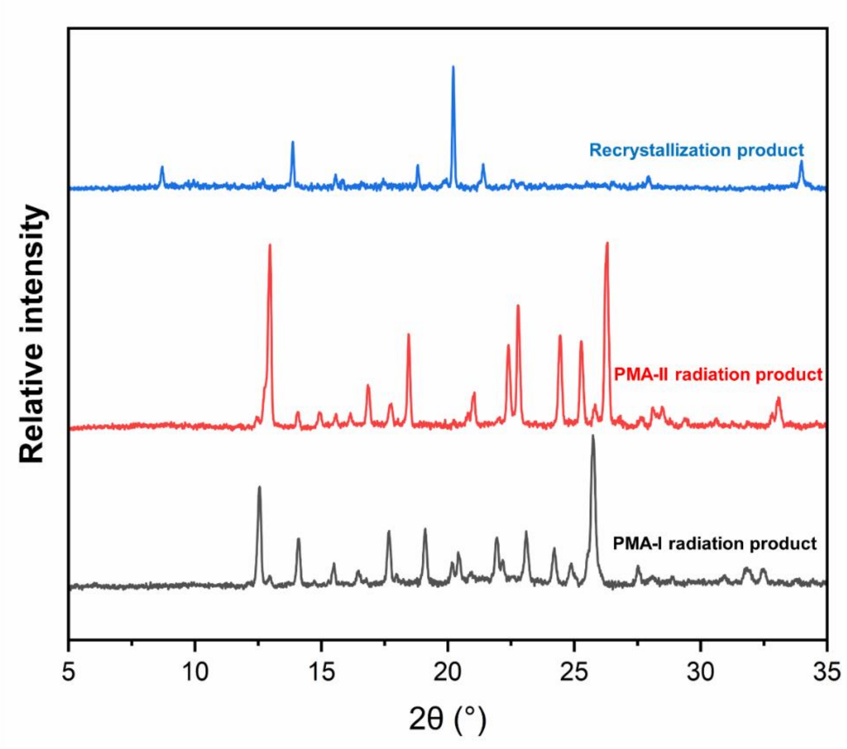

Supplementary Figure 9. PXRD patterns of the products obtained by irradiation of PAM-I and PMA-II, and after recrystallization.

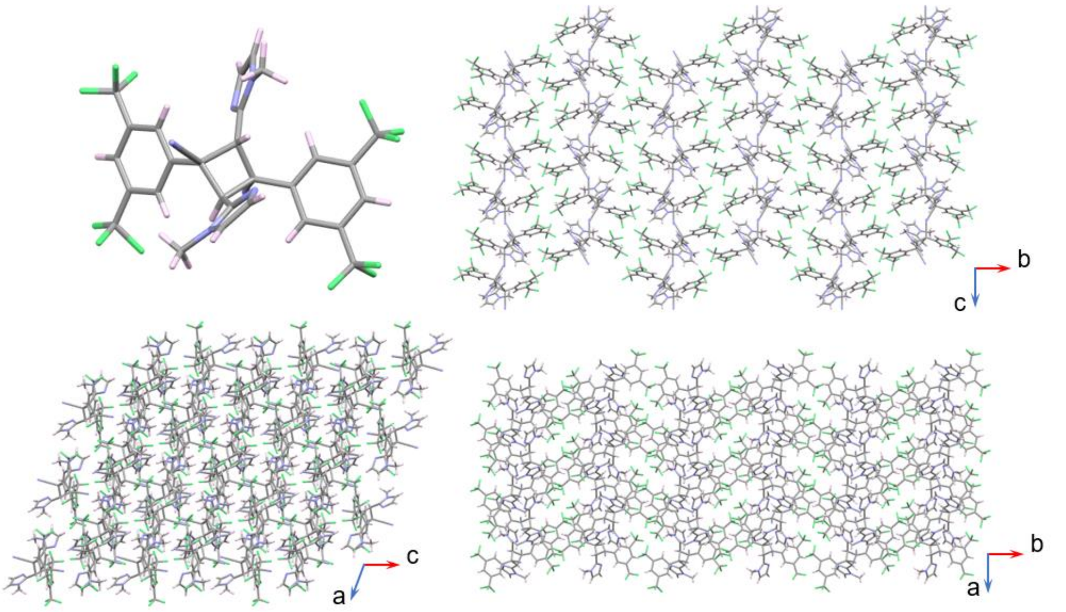

Supplementary Figure 10. Packing arrangement of the [2 + 2] photocycloaddition product.

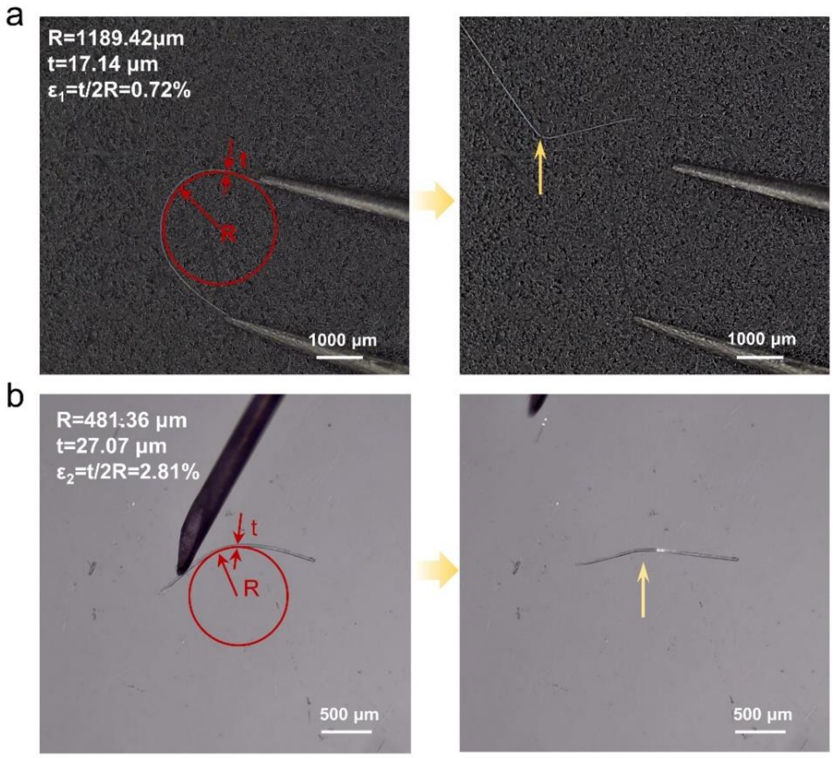

Supplementary Figure 11. Mechanical response of crystals of PMA-III. (a) bending along the (001) plane. (b) bending along the (010) plane. In this figure, R is the radius of curvature. In (a) t is the thickness of (010) plane, and in (b) t is the thickness of (001) plane. ε_n is the bending strain.

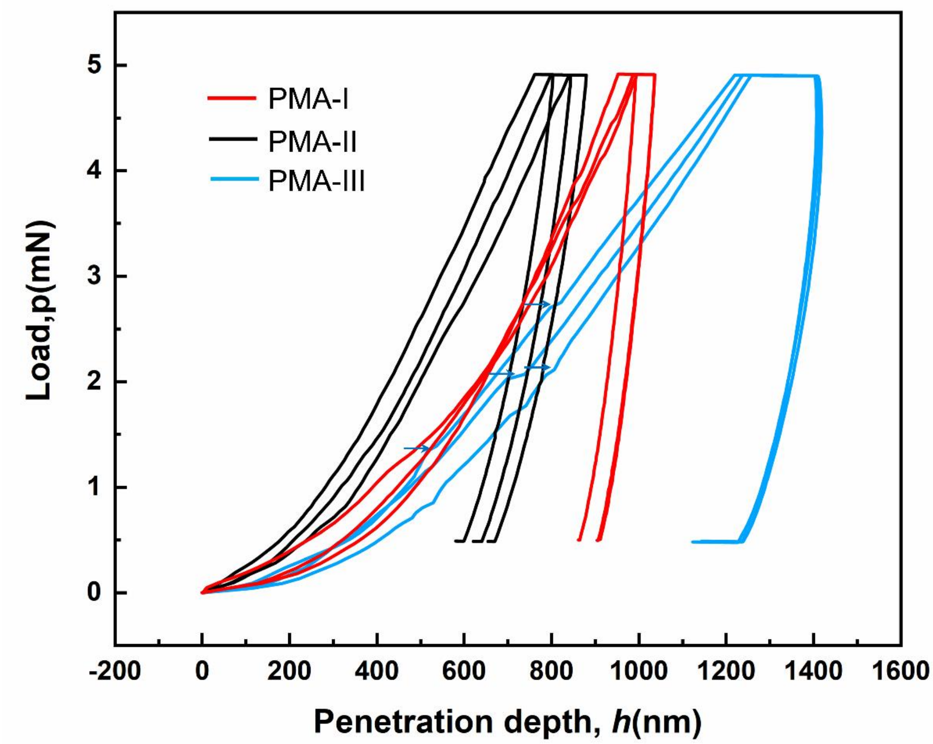

Supplementary Figure 12. Load-depth ($P-h$) curves obtained by nanoindentation on the wide facets of crystals of PMA-I, PMA-II and PMA-III.

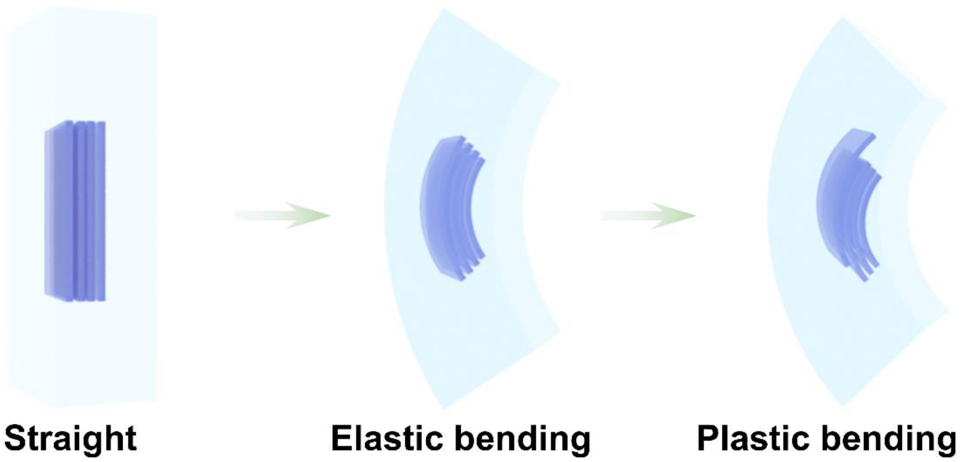

Supplementary Figure 13. Schematic diagram of the suggested bending mechanism of a crystal of PMA-III.

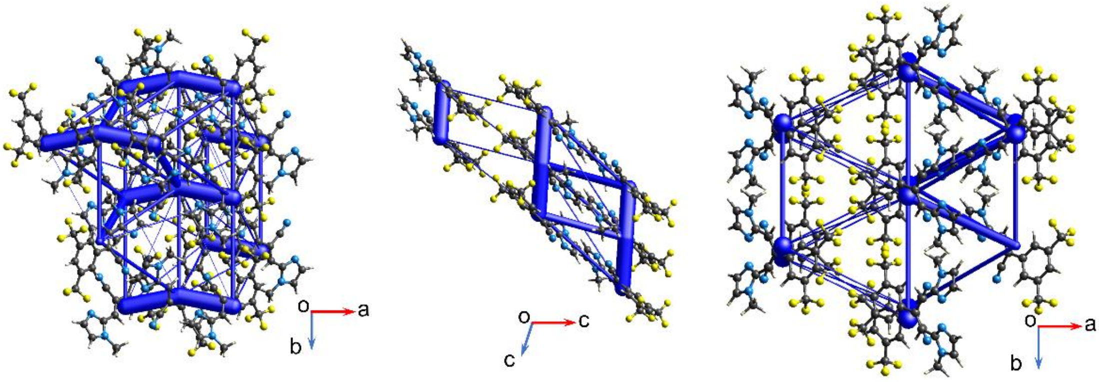

Supplementary Figure 14. Energy frameworks of the polymorph PMA-III viewed along different crystallographic axes.

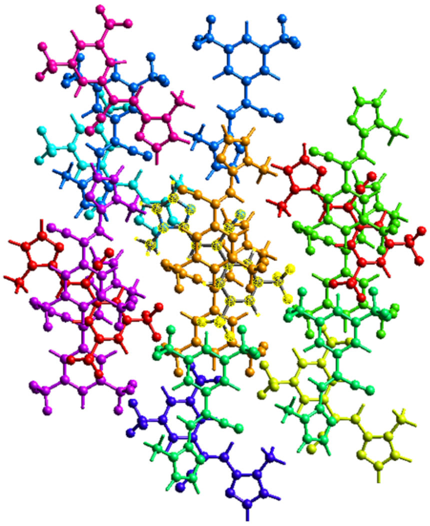

Interaction Energies (kJ/mol)
R is the distance between molecular centroids (mean atomic position) in Å.

Total energies, only reported for two benchmarked energy models, are the sum of the four energy components, scaled appropriately (see the scale factor table below)

	N	Symop	R	Electron Density	E_ele	E_pol	E_dis	E_rep	E_tot
	2	x, y, z	9.25	B3LYP/6-31G(d,p)	-7.1	-2.3	-12.0	2.8	-17.8
	2	x, -y+1/2, z+1/2	4.61	B3LYP/6-31G(d,p)	-27.9	-7.9	-68.5	36.4	-72.5
	1	-x, -y, -z	10.78	B3LYP/6-31G(d,p)	0.6	-0.1	-7.6	0.9	-5.6
	2	x, -y+1/2, z+1/2	9.99	B3LYP/6-31G(d,p)	2.4	-0.4	-6.1	0.4	-2.8
	2	-x, y+1/2, -z+1/2	9.45	B3LYP/6-31G(d,p)	0.2	-0.1	-7.9	0.9	-6.2
	1	-x, -y, -z	10.90	B3LYP/6-31G(d,p)	-13.7	-4.2	-30.4	13.2	-35.9
	2	-x, y+1/2, -z+1/2	12.62	B3LYP/6-31G(d,p)	-9.2	-5.5	-15.4	18.4	-15.8
	1	-x, -y, -z	11.10	B3LYP/6-31G(d,p)	-0.3	-0.1	-7.1	0.8	-6.1
	2	x, -y+1/2, z+1/2	10.66	B3LYP/6-31G(d,p)	2.9	-1.7	-8.5	4.7	-2.6
	1	-x, -y, -z	15.77	B3LYP/6-31G(d,p)	-4.3	-0.3	-2.6	0.7	-6.7

Scale factors for benchmarked energy models
See Mackenzie et al. IUCrJ (2017)

Energy Model	k_ele	k_pol	k_disp	k_rep
CE-HF ... HF/3-21G electron densities	1.019	0.651	0.901	0.811
CE-B3LYP ... B3LYP/6-31G(d,p) electron densities	1.057	0.740	0.871	0.618

Supplementary Figure 15. The total energies of PMA-I based on B3LYP/6-31G(d,p) calculated using CrystalExplorer. The packing diagram is viewed down the *c* axis.

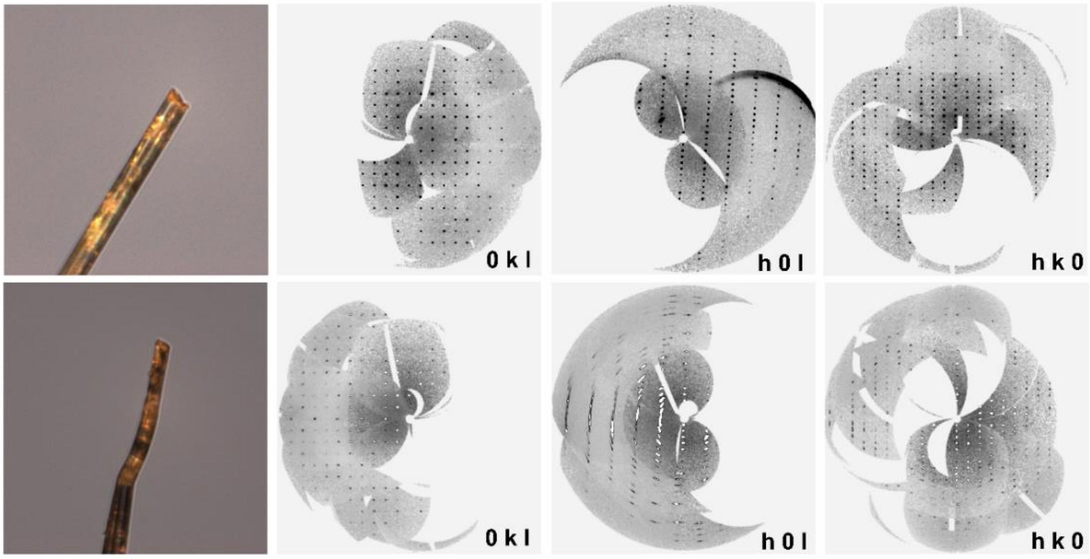

Supplementary Figure 16. X-ray diffraction patterns of straight and bent crystals of PMA-III.

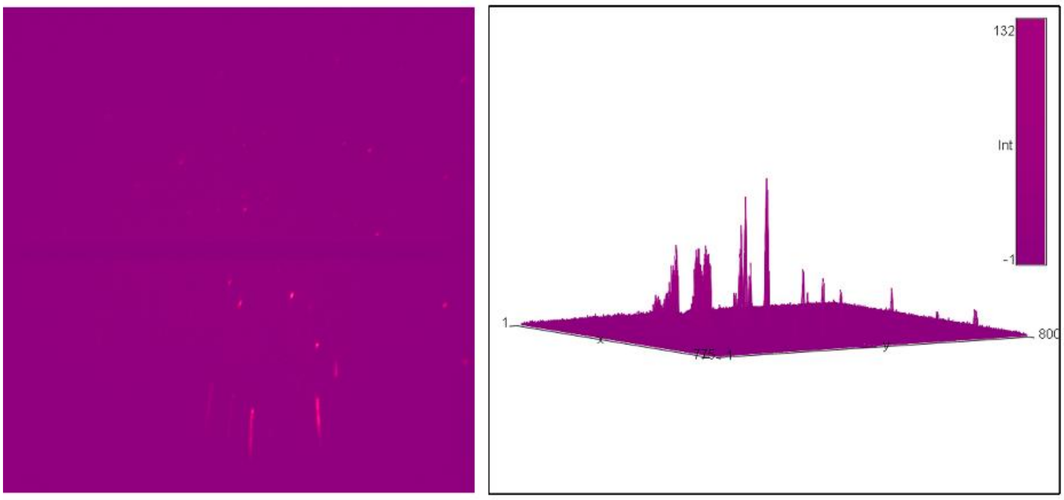

Supplementary Figure 17. Exemplary images of the Bragg diffraction peaks of bent crystals.

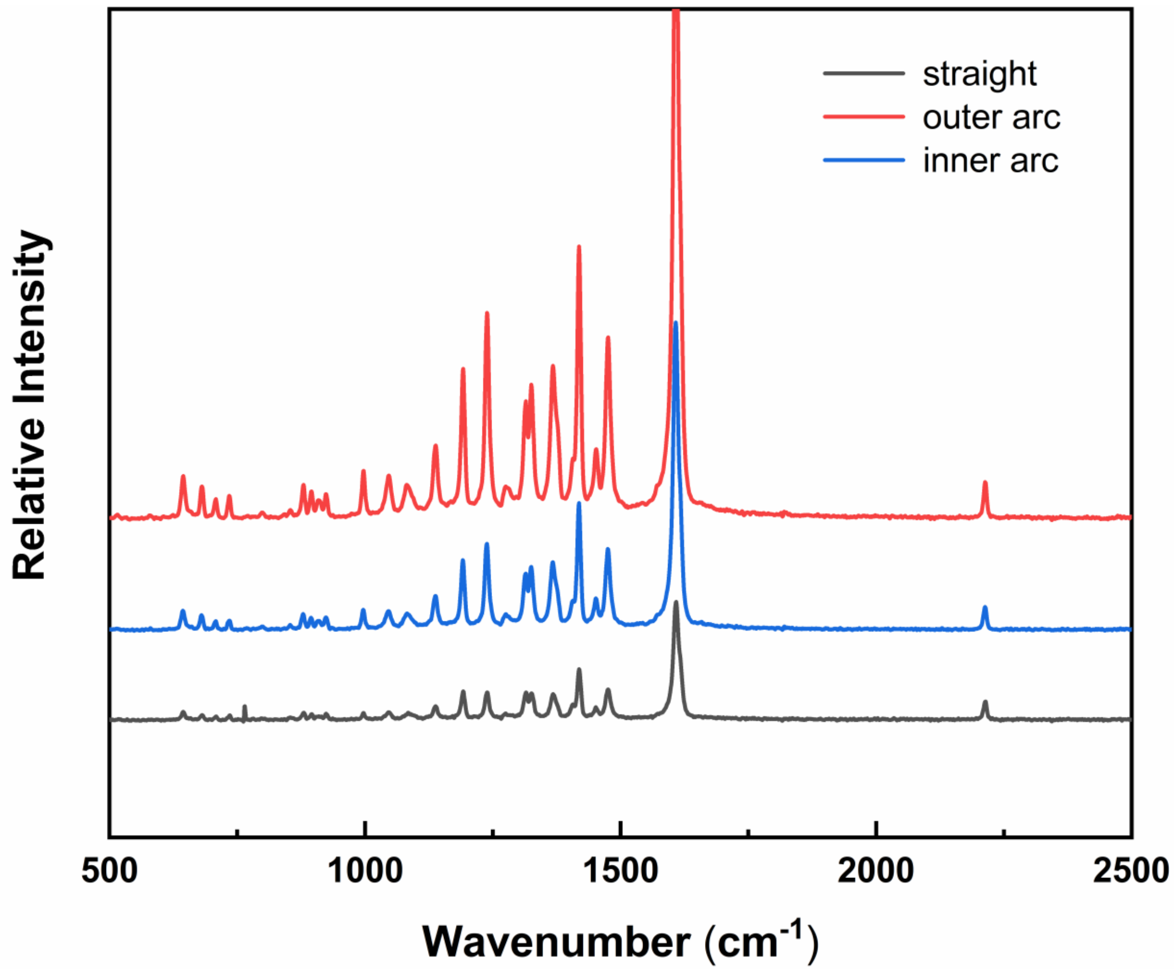

Supplementary Figure 18. Raman spectra of straight and bent crystals recorded along the (010) plane.

Supplementary Tables

Supplementary Table 1. Crystallographic data for PMA and its photoproduct (TMC)

	PMA- I	PMA- II	PMA- III	TMC
**Crystal system**	Orthorhombic	Monoclinic	Monoclinic	Monoclinic
**Temperature/ K**	113.15	113.15	277.09	159.99
**Space group**	*Pbca*	$P2_1/c$	$P2_1/c$	$P2_1/n$
Z	8	4	4	4
**Formula weight**	345.25	345.25	345.25	690.50
**Color**	green	colorless	colorless	colorless
**a**	7.9407(3)	15.4734(9)	17.5836(3)	10.0905(4)
**b**	12.8084(5)	7.8129(3)	9.2458(2)	24.4237(8)
**c**	28.4658(9)	13.1933(6)	9.1922(2)	12.9626(5)
**α**	90	90	90	90
**β**	90	114.316(6)	93.653(2)	107.872(4)
**γ**	90	90	90	90
**V**	2895.19	1453.47	1491.38	3040.44
**Density/ (g/cm^3)**	1.584	1.578	1.538	1.508

F_{000}	1392	696	696	1392
h_{min}, h_{max}	-12,12	-19,19	-21,22	-11,12
k_{min}, k_{max}	-18,18	9, -9	-11,11	-21,30
l_{min}, l_{max}	-43,43	-16,13	-8,10	-16,16
**No. of measured reflections**	41249	17043	9674	20689
**No. of unique reflections**	5092	2966	2950	6029
**No. of reflections used**	3873	2100	2545	4798
**No. of refinement parameters**	219	363	274	545
**CCDC number**	2278400	2278402	2278403	2278404

Supplementary Table 2. Geometric parameters of PMA-I and PMA-II relevant to dimerization

	d (Å)	θ_1 (°)	θ_2 (°)	θ_3 (°)
**Ideal**	＜4.2	0	90	90
**PMA-I**	4.045	0	95.65	56.29
**PMA-II**	3.726	0	90.64	67.61
**PMA-III**	4.972	42.04	103.56	46.46

Supplementary Table 3. Young's modulus and hardness of the polymorphs based on nanoindentation

	Modulus/GPa	Hardness/GPa
**PMA-I**	10.15 ± 0.53	0.21 ± 0.01
**PMA-II**	8.97 ± 0.59	0.36 ± 0.03
**PMA-III**	7.45 ± 0.18	0.11 ± 0.005

Supplementary Table 4. Results from the measurement of the actuation performance (# refers to the sample number

#	Prismatic crystals		Acicular crystals	
	Work density/ J m^{-3}	Force density/ N m^{-3}	Work density/ J m^{-3}	Force density/ N m^{-3}
1	3.12E+03	3.37E+07	7.84 E+01	1.68E+06
2	1.55E+03	3.87E+07	1.23 E+02	1.80E+06
3	1.98E+03	4.59E+07	1.14 E+02	2.49E+06
4	1.62E+03	5.04E+07	2.82 E+02	2.86E+06
5	2.67E+03	5.18E+07	2.56 E+02	3.17E+06
6	4.80E+03	5.42E+07	1.08 E+02	3.32E+06
7	2.77E+03	5.42E+07	7.67 E+01	3.34E+06
8	4.79E+03	5.88E+07	2.53 E+02	4.01E+06
9	4.20E+03	6.07E+07	2.52 E+02	4.31E+06
10	3.63E+03	7.22E+07	1.32 E+02	4.62E+06
11	1.53E+03	7.26E+07	7.04 E+01	4.98E+06
12	3.25E+03	7.53E+07	6.02 E+01	5.21E+06
13	4.49E+03	8.47E+07	1.25 E+02	7.20E+06
14	6.47E+03	8.86E+07	7.81 E+02	9.48E+06

15	2.62E+03	1.09E+08	5.27 E+02	1.31E+07
16	5.47E+03	1.09E+08	8.90 E+02	1.65E+07
17	2.37E+03	1.10E+08	1.07 E+03	1.81E+07
18	3.01E+03	1.22E+08	1.08 E+03	2.07E+07
19	6.60E+03	1.43E+08	3.09 E+03	2.32E+07
20	1.37E+04	1.68E+08	4.38 E+02	2.35E+07
21	5.18E+03	1.82E+08	1.31 E+03	3.64E+07
22	9.50E+03	2.03E+08	2.93 E+03	3.71E+07
23	1.62E+04	2.55E+08	2.31 E+03	4.39E+07
24	1.06E+04	2.80E+08	2.73 E+03	4.66E+07
25	7.04E+03	3.86E+08	1.50 E+03	4.67E+07
26	2.77E+04	3.88E+08	8.76 E+02	4.70E+07
27	2.85E+04	3.97E+08	1.44 E+03	4.72E+07
28	2.53E+04	4.23E+08	2.60 E+03	4.95E+07
29	2.00E+04	5.11E+08	2.03E+03	5.40E+07
30	4.43E+03	5.39E+08	1.28 E+03	6.43E+07
31	1.56E+04	5.46E+08	3.48 E+03	6.61E+07
32	2.48E+04	5.82E+08	2.11 E+03	7.19E+07
33	2.34E+04	5.93E+08	3.94 E+03	7.24E+07
34	1.98E+04	6.59E+08	3.05 E+03	8.18E+07
35	2.58E+04	6.59E+08	1.66 E+03	8.98E+07
