# Supplementary material for: Highly efficient in crystallo energy transduction of light to work
Source: Nat Commun. 2024 Apr 29;15:3633. doi: 10.1038/s41467-024-47881-6 (PMC11059232; doi:10.1038/s41467-024-47881-6)
Supplement: Supplementary file 3 — Description of Additional Supplementary Files [file 41467_2024_47881_MOESM3_ESM.pdf]

Description of Additional Supplementary Files

File Name: Supplementary Movie 1
Description: Photosalient effects of prismatic PMA-I

File Name: Supplementary Movie 2
Description: High-speed recordings of the photosalient effects of PMAI

File Name: Supplementary Movie 3
Description: Photoinduced bending of acicular PMA-I

File Name: Supplementary Movie 4
Description: Photoinduced rolling of PMA-II

File Name: Supplementary Movie 5
Description: Mechanically induced bending along the (001) plane of PMA-III

File Name: Supplementary Movie 6
Description: Mechanically induced bending along the (010) plane of PMA-III

File Name: Supplementary Movie 7
Description: Steel ball displacement by deformation of acicular crystal of PMA-I

File Name: Supplementary Movie 8
Description: Glass plate displacement by disintegration of a prismatic crystal of PMA-I
